# Ilizarov external fixation versus plate internal fixation in the treatment of end-stage ankle arthritis: decision analysis of clinical parameters

**DOI:** 10.1038/s41598-017-16473-4

**Published:** 2017-11-23

**Authors:** Jun Li, Bohua Li, Zhengdong Zhang, Shanxi Wang, Lei Liu

**Affiliations:** Department of Orthopedics, West China Hospital, Sichuan University, 37# Wainan Guoxue Road, Chengdu, 610041 People’s Republic of China

## Abstract

The purpose of this study was to evaluate the effect of Ilizarov external fixation (IEF) and plate internal fixation (PIF) in the treatment of end-stage ankle arthritis on pain relieving and function improvement. The study cohort consisted of 59 patients with end-stage ankle arthritis underwent ankle arthrodesis with IEF or PIF between June 2011 and June 2015. Standard radiographs and computed tomography (CT) scans were obtained before surgery and during the follow-up. Functional assessments were performed using Foot and Ankle pain score of American Orthopedics Foot and Ankle Society (AOFAS) and Visual Analogue Scale (VAS). The average AOFAS scores in both IEF group and PIF groups increased significantly after operation, from 45.5 ± 6.3 to 84.8 ± 4.9 and from 45.9 ± 6.6 to 86.6 ± 5.4, respectively. The average VAS scores in both groups decreased significantly after operation, from 8.4 ± 1.9 to 2.5 ± 0.6 and from 8.2 ± 1.5 to 2.3 ± 0.7, respectively. Nevertheless, there was no significant difference for preoperative or postoperative AOFAS and VAS scores between the two groups. The IEF would result in comparable postoperative functional recovery and pain relieving to PIF and may be an effective substitute to PIF in the treatment of end-stage ankle arthritis.

## Introduction

The consequences of end-stage ankle arthritis incorporate not only inferior ankle joint function but also poor general physical condition, which can significantly lower the quality of life for its victims^1–3^. Operative treatment for end-stage ankle arthritis is usually demanding and difficult on account of the restrictions caused by the etiology and the patient’s condition^4–6^. Arthrodesis is often a limb salvage procedure in patients with end-stage ankle arthritis that may warrant a below-knee amputation as the only alternative^7^. More than forty techniques have been recorded in the literature for arthrodesis, including crossed screw structure, intramedullary nail, plate, external fixation frame and so forth, yet there remains much controversy with respect to the optimal technique for ankle fusion to acquire steady rigid fixation accompanying restoration of plantigrade foot function^8–11^. Furthermore, existing techniques are associated with several complications such as malalignment, infection, nonunion, and adjacent joint osteoarthritis^12^.

Both internal and external fixations have demonstrated positive results in achieving ankle fusion^13^. Superiority of PIF incorporates ready availability of fixation materials, comparatively inexpensive, convenience in applying, and verified therapeutic benefits under applicative conditions of patients^14^. Compared with IEF, PIF is associated with shorter operative time, greater patient satisfaction and shrinking complications^15^. Nevertheless, there are several conditions where appliance of the IEF provides obvious advantages over PIF, such as peripheral vascular disease, diabetes mellitus, rheumatoid disease and Charcot disease^16,17^. Besides, It can provides adequate dynamic axial compression of flat and allows the surgeon to address any intraoperative errors or early postoperative loss of position^18^. The merits and demerits of each arthrodesis strategy should be taken into account when determining the process of management to achieve the best clinical outcome.

To the best of our knowledge, study that focus on comparison of IEF and PIF in the treatment of end-stage ankle arthritis is still missing. Therefore, the purpose of our study was (1) to compare the effect of IEF and PIF in the treatment of end-stage ankle arthritis on pain relieving, deformity correction, and function improvement, and (2) provide a reference for future management of end-stage ankle arthritis.

## Patients and Methods

This retrospective study was conducted at the Department of Orthopedic Surgery at West China Hospital, Sichuan University and was approved by the internal research administration department and ethical committee. Written informed consent from all the participants was obtained. The clinical study was conducted in accordance with the Declaration of Helsinki principles. The medical records and radiographs of patients with end-stage ankle arthritis treated at our institution were reviewed by two orthopedic surgeons who did not participate in the operation. The ankle arthritis etiology and latency time from initial injury or diagnosis to the index surgery were recorded. Inclusion criteria were as follows: symptomatic end-stage ankle arthritis, failure of at least a year of non-operative treatment, age between 18 and 60 years, unilateral ankle affected, treated with IEF or PIF. Exclusion criteria included bilateral lesions, an age younger than 18 years or older than 60 years, severe systemic illness, current alcohol or drug abuse.

After obtaining the consent from our Institutional Review Board, we retrospectively reviewed a continuous series of end-stage ankle arthritis treated at our hospital between June 2011 and June 2015. During the data collection period, 64 patients with end-stage ankle arthritis met the inclusion criteria. Another 5 patients were also excluded because of the follow-up less than 24 months. Finally, 59 patients were incorporated into the study cohort, among which 31 were treated with IEF and 28 PIF.

**Figure 1:**
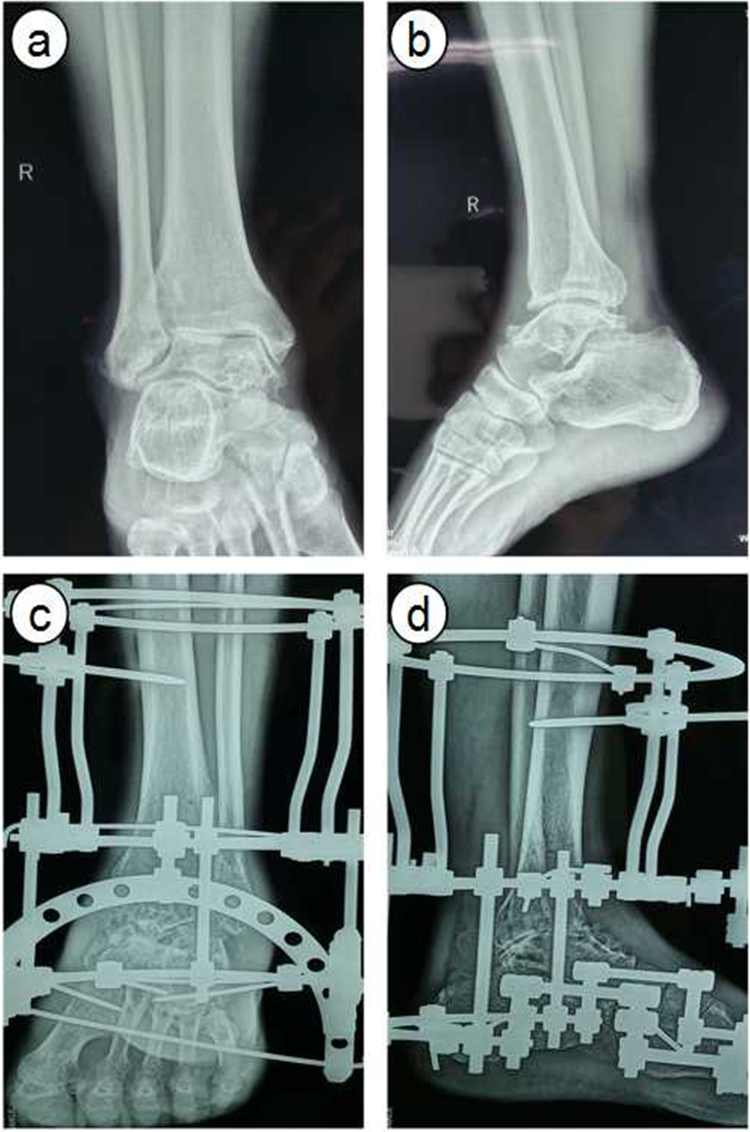
Radiographs showing a 57 yrs patient with end-stage ankle arthritis underwent Ilizarov external fixation. (**a**) and (**b**) pre-operative anteroposterior and lateral radiographs, (**c**) and (**d**), post-operative 3 month anteroposterior and lateral radiographs.

All operations were performed under general anesthesia by a senior surgeon. For patients undergoing IEF, the circular frame was installed according to the order of tibia, ankle and foot (Fig. 1). After the rotation center of ankle being attached to the rotation center of external frame and shank being placed in the middle of the ring, a k-wire was pierced through the distal tibia. Two crossed k-wire were pierced through the middle and lower tibia to fix the proximal frame. After the rotation center being adjusted, a k-wire was pierced through middle lower section of 1–5 metatarsal from inside to outside. A k-wire was driven perpendicular to calcaneus from inside to outside to fix the supporting ring of foot. Two half wire were driven behind the calcaneus evading the achilles tendon and a k-wire was driven from the inside of first metatarsal. The detached cancellous bone was crushed and filled in the space of ankle joint, then covered with gelatin sponge. After external frame being pressed, the ankle joint was fixed at functional position.

**Figure 2:**
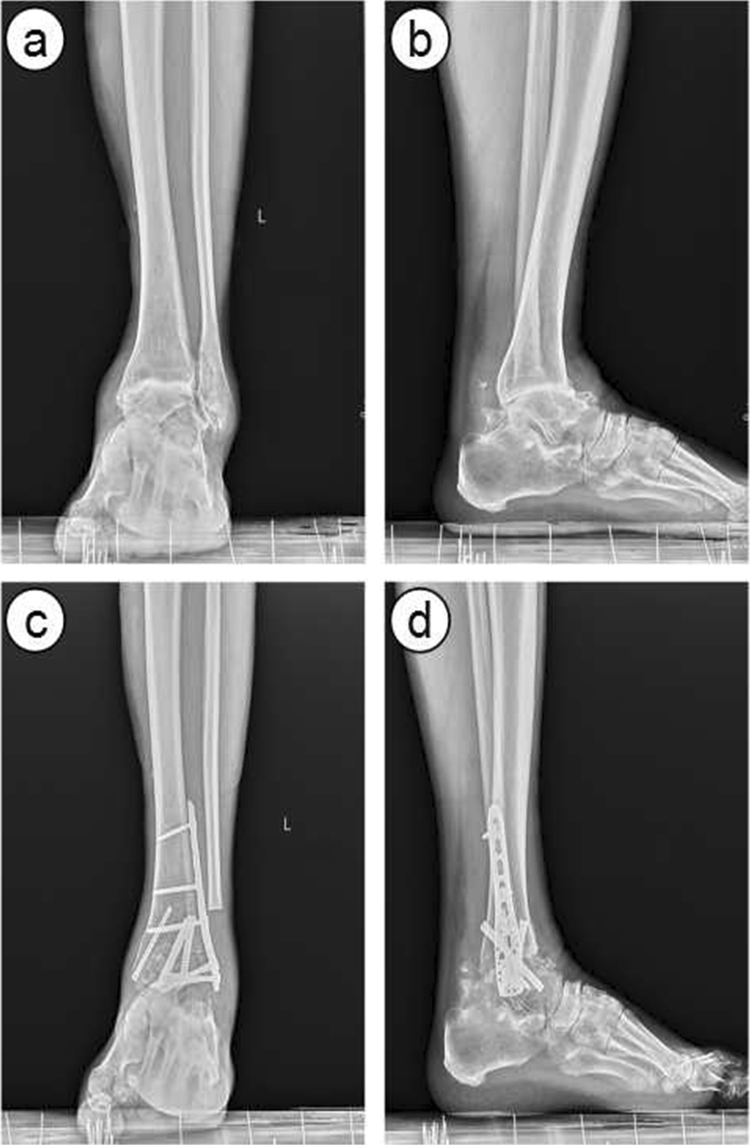
Radiographs showing a 45 yrs patient with end-stage ankle arthritis underwent plate internal fixation. (**a**) and (**b**) pre-operative anteroposterior and lateral radiographs, (**c**) and (**d**), post-operative 3 month anteroposterior and lateral radiographs.

For patients undergoing PIF, a locking compression plate with appropriate length was placed at the distal tibia, the talus dorsal bone was then pruned to make sure the steel plate attachment is good (Fig. 2). Two screws were screwed into the distal side to maintain the fusion position of tibiotalar joint, the interface of fusion bone was pressed using a specially designed pressure sliding hole in the plate. Two screws were screwed into the proximal side and then the rest. The ankle joint was fixed at functional position. Make sure no less than three screws were set in each side of the plate.

To avoid examiner bias, clinical evaluations and postoperative follow-up were performed by two independent surgeons who were not involved in the surgical treatment of the patients. Standard radiographs and CT scans were obtained before surgery and during the follow-up. Patients were also inquired about their overall experience, real-time status of their ankle, complications and subsequent treatments. Effectiveness of operation was evaluated using Foot and Ankle pain score of American Orthopedics Foot and Ankle Society (AOFAS) and Visual Analogue Scale (VAS).

Statistical analysis was performed using Statistical Package for the Social Sciences (SPSS 19.0, IBM, NYC, USA). Categorical data were tabulated with frequencies or percentages and continuous data were expressed as mean ± Standard Deviation (SD). Normality was tested using the Kolmogorov-Smirnov test. The chi-square test for categorical variables was used to compare patients’ characteristics at baseline. The independent t test was used to analyze inter-group comparisons for normally distributed continuous data, and the Mann-Whitney U tests was used to analyze skewed data. The paired t test and Wilcoxon rank test were used to compare intra-group continuous variables with or without normal distribution, respectively. P value of less than 0.05 was considered statistically significant.

## Results

### Baseline characteristics

The baseline characteristics of the patients in both groups are summarized in Table 1. Fifty nine patients with end-stage ankle arthritis underwent ankle arthrodesis with IEF (n = 31, 17 males [54.8%] and 14 females [45.2%]) or PIF (n = 28, 16 males [57.1%] and 12 females [42.9%]). There was no significant difference in age, gender, affected side, body mass index (BMI), duration of symptoms, and follow-up duration between the IEF and PIF groups (P > 0.05 for all parameters). The main cause was traumatic arthritis (61.3% and 60.7% respectively), followed by osteoarthritis (19.4% and 17.9% respectively) and rheumatoid arthritis (12.9% and 14.3%). Other reasons included paralytic, Charcot arthropathy, or talus necrosis.

**Table 1:** Comparison of the baseline data of the patients between the IEF and PIF group.

	IEF group (*n* = 31)	PIF group (*n* = 28)	*P* value*
Age (years)	45.6 ± 8.9	46.8 ± 9.1	0.6109
Gender (male/female)	17/14	16/12	0.8587
BMI (kg/m^2^)	24.8 ± 3.7	25.2 ± 3.9	0.6875
Side (left/right)	13/18	11/17	0.8361
Duration of symptoms (months)	11.7 ± 4.6	12.4 ± 5.5	0.5968
Follow-up duration (months)	29.2 ± 3.5	28.4 ± 3.0	0.3524
Causes (%)			0.9822
Traumatic arthritis	19 (61.3%)	17 (60.7%)	
Osteoarthritis	6 (19.4%)	5 (17.9%)	
Rheumatoid arthritis	4 (12.9%)	4 (14.3%)	
Other	2 (6.4%)	2 (7.1%)	

IEF (Ilizarov external fixation), and PIF (plate internal fixation), BMI (Body mass index). *Independent *t* test or chi-square *t*est. The *P* values shown are for inter-group comparisons.

### Clinical outcomes

The clinical outcomes between the two groups are listed in Table 2. The IEF needed longer operative time compared with PIF (104.7 ± 13.8 vs. 73.6 ± 11.2 min, P < 0.0001). The IEF group gave rise to more perioperative blood loss than that of PIF group (146.8 ± 36.2 vs. 105.9 ± 27.5 ml, P < 0.0001). However, the duration of bony fusion of IEF group was significantly shorter than that of PIF group (12.1 ± 2.3 vs. 17.5 ± 2.9 weeks, P < 0.0001). The final complete fusion rates in IEF group and PIF group were 100% and 96.4%, respectively.

**Table 2:** Comparison of clinical outcomes between the IEF and PIF group.

	**IEF group** (*n* = **31)**	**PIF group** (*n* = **28)**	*P* value*
Operative time (min)	104.7 ± 13.8	73.6 ± 11.2	<0.0001
Blood loss (ml)	146.8 ± 36.2	105.9 ± 27.5	<0.0001
Duration of fusion (weeks)	13.0 ± 2.3	17.5 ± 2.9	<0.0001
Fusion rate (%)	31/31 (100%)	27/28 (96.4%)	0.9218
**AOFAS score**			
Preoperative	45.5 ± 6.3	45.9 ± 6.6	0.8127
Final follow-up	84.8 ± 4.9	86.6 ± 5.4	0.1848
**VAS score**			
Preoperative	8.4 ± 1.9	8.2 ± 1.5	0.6577
Final follow-up	2.5 ± 0.6	2.3 ± 0.7	0.2423
Complications			0.7304
Midfoot pain	3 (9.7%)	2 (7.1%)	
Infection	1 (3.2%)	2 (7.1%)	
Nonunion	0 (0)	1 (3.6%)	

*Independent *t* test or chi-square test. The *P* values shown are for inter-group comparisons.

The average AOFAS scores in both IEF group and PIF groups increased significantly after operation, from 45.5 ± 6.3 to 84.8 ± 4.9 and from 45.9 ± 6.6 to 86.6 ± 5.4, respectively. The average VAS scores in both groups decreased significantly after operation, from 8.4 ± 1.9 to 2.5 ± 0.6 and from 8.2 ± 1.5 to 2.3 ± 0.7, respectively. Nevertheless, there was no significant difference for preoperative or postoperative AOFAS and VAS scores between the two groups (P > 0.05).

During the follow-up, three patients (9.7%) in the IEF group and two patients (7.1%) in the PIF group appeared varying degree of midfoot pain, which was managed by receiving nonsteroidal anti-infammatory drugs(NSAIDs). There was one case (3.2%) in the IEF group and two cases (7.1%) in the PIF group with superficial wound infections, which were settled by wound dressing. No bone nonunion occurred in the IEF group, while one nonunion happened in the PIF group due to plate breakage, which was successfully treated by revision surgery. No severe complications occurred in both groups, for instance, deep infection, nerve injury, and deep vein thrombosis.

## Discussion

The present study retrospectively analyzed the outcomes and complications of 59 patients with end-stage ankle arthritis and received ankle arthrodesis surgery through IEF or PIF. Compared with PIF, the IEF required longer operative time and induced more perioperative blood loss, however, it needed less postoperative fusion time than PIF. At final follow-up, the average AOFAS score in IEF group was slightly lower than that of PIF group, but the difference was not statistically significant.

Although internal fixation is currently the most widely used method for ankle arthrodesis, the impact of other fixation techniques is becoming increasingly apparent with the increase of complex ankle lesions^6,19^. In comparison to internal plates and intramedullary nails, Ilizarov external fixators induce less disruption to the soft tissues, periosteum, and blood supply of bone^20^. This property makes IEF ideal for soft tissue management in patients with poor skin conditions whose rehabilitation potential is compromised as in the cases of peripheral vascular disease, diabetes mellitus, Charcot disease, and rheumatoid disease^3,21^. Ilizarov external fixators are fixed angle devices and can provide more rigid fixation in compromised bone^22^. The ability to use these fixed angle wires in multiple flats enables the versatility required to optimize steadiness while minimizing tissue damage^23^.

Achieving arthrodesis in patients with ankle destruction and other comorbidities, such as immune deficiency, diabetes mellitus, malnutrition, renal/liver failure, extensive scarring, chronic lymphedema, major vessel disease, smoking, chronic hypoxia, or malignancy, presents a reconstructive challenge to the orthopaedic surgeon^24,25^. It is difficult for these patients to get the bony healing and usually leave them no choice but conservative management or amputation^26^. IEF has been regarded as a last resort therapy for limb salvage in the disposal of these complex cases^16,27^. Kugan *et al*.^28^ achieved an 83% fusion rate with satisfying functional improvement and no recurrence of previous deep infection in 48 patients with multiple comorbidities using the Ilizarov technique alone. Despite a few expected complications, most of which can be cured with early detection, IEF serves as a reasonable limb salvage alternative to amputation in this subset of patients^29^.

The present study had several limitations. It is a single-center retrospective analysis. There was no randomization. The sample size was relatively small. A prospective randomized controlled trial involving more cases is needed in the future. However, the consequences of this study could provide reference for the management of end-stage ankle arthritis.

In conclusion, the IEF would result in comparable postoperative functional recovery and pain relieving to PIF, as determined by AOFAS score, VAS score, and fusion rate as well as complication rates. These results suggest that Ilizarov external fixation may be an effective substitute to plate internal fixation in the treatment of end-stage ankle arthritis.

### Data availability statement

The datasets generated and analysed during the current study are available from the corresponding author on reasonable request.
